# Actions of a Novel Bacterial Topoisomerase Inhibitor against *Neisseria gonorrhoeae* Gyrase and Topoisomerase IV: Enhancement of Double-Stranded DNA Breaks

**DOI:** 10.3390/ijms241512107

**Published:** 2023-07-28

**Authors:** Soziema E. Dauda, Jessica A. Collins, Jo Ann W. Byl, Yanran Lu, Jack C. Yalowich, Mark J. Mitton-Fry, Neil Osheroff

**Affiliations:** 1 Department of Biochemistry, Vanderbilt University School of Medicine, Nashville, TN 37232, USA; 2 Division of Medicinal Chemistry and Pharmacognosy, College of Pharmacy, The Ohio State University, Columbus, OH 43210, USA; 3 Division of Pharmaceutics and Pharmacology, College of Pharmacy, The Ohio State University, Columbus, OH 42310, USA; 4 Department of Medicine (Hematology/Oncology), Vanderbilt University School of Medicine, Nashville, TN 37232, USA; 5 VA Tennessee Valley Healthcare System, Nashville, TN 37212, USA

**Keywords:** novel bacterial topoisomerase inhibitor, NBTI, type II topoisomerase, gyrase, topoisomerase IV, DNA cleavage, *Neisseria gonorrhoeae*

## Abstract

Novel bacterial topoisomerase inhibitors (NBTIs) are an emerging class of antibacterials that target gyrase and topoisomerase IV. A hallmark of NBTIs is their ability to induce gyrase/topoisomerase IV-mediated single-stranded DNA breaks and suppress the generation of double-stranded breaks. However, a previous study reported that some dioxane-linked amide NBTIs induced double-stranded DNA breaks mediated by *Staphylococcus aureus* gyrase. To further explore the ability of this NBTI subclass to increase double-stranded DNA breaks, we examined the effects of OSUAB-185 on DNA cleavage mediated by *Neisseria gonorrhoeae* gyrase and topoisomerase IV. OSUAB-185 induced single-stranded and suppressed double-stranded DNA breaks mediated by *N. gonorrhoeae* gyrase. However, the compound stabilized both single- and double-stranded DNA breaks mediated by topoisomerase IV. The induction of double-stranded breaks does not appear to correlate with the binding of a second OSUAB-185 molecule and extends to fluoroquinolone-resistant *N. gonorrhoeae* topoisomerase IV, as well as type II enzymes from other bacteria and humans. The double-stranded DNA cleavage activity of OSUAB-185 and other dioxane-linked NBTIs represents a paradigm shift in a hallmark characteristic of NBTIs and suggests that some members of this subclass may have alternative binding motifs in the cleavage complex.

## 1. Introduction

**Figure 1:**
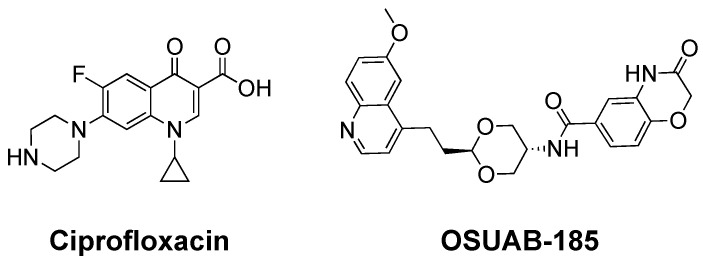
Structures of the fluoroquinolone ciprofloxacin and the NBTI OSUAB-185.

Fluoroquinolones, such as ciprofloxacin (Figure 1) and moxifloxacin, are among the most efficacious and broad-spectrum oral antibacterials in clinical use [1,2,3]. The World Health Organization (WHO) lists fluoroquinolones in their five “Highest Priority Critically Important Antimicrobials”, and these drugs are heavily used worldwide [1,3].

There is a growing crisis in antibacterial resistance. Unfortunately, fluoroquinolone resistance is becoming prevalent and is impeding the clinical efficacy of this important drug class [2,4,5,6,7]. For example, ciprofloxacin was used as frontline treatment for gonorrheal infections in humans starting in 1993 [8]. By 2003, >40% of cases of gonorrhea were treated with fluoroquinolones [9]. However, their use as routine therapy was discontinued in 2006 due to the high incidence of resistance (>30% of gonorrhea cases in the United States are currently resistant to fluoroquinolones) [10,11]. Drug-resistant *Neisseria gonorrhoeae*, the causative agent of gonorrhea, is categorized by the Centers for Disease Control (CDC) as one of the five “urgent level” antibiotic resistance threats in the United States [12]. It is a major cause of pelvic inflammatory disease and infertility and appears to facilitate the transmission of HIV [13,14]. Current estimates suggest new cases of gonorrhea exceed 82 million annually, and the WHO has issued dire warnings that gonorrhea has the potential to join herpes and HIV/AIDS as the third “incurable” sexually transmitted disease [15,16].

The targets for fluoroquinolones are the bacterial type II topoisomerases, gyrase and topoisomerase IV [4,5,6,7]. These enzymes modulate the superhelical state of the bacterial chromosome, remove positive supercoils that accumulate ahead of replication forks and transcription complexes, and remove tangles and knots from the genome [17,18,19,20,21,22,23]. Gyrase and topoisomerase IV regulate DNA topology by passing an intact double helix through a transient break that they generate in a second DNA segment [19,21,24,25,26]. To maintain genomic integrity while the DNA is cut, the enzymes form covalent bonds between active-site tyrosine residues and the newly generated 5′-DNA termini. These covalent enzyme-cleaved DNA complexes are known as “cleavage complexes” [4,19,21,23,25,26]. Fluoroquinolones kill bacterial cells by inserting into the cleaved DNA scissile bonds on both strands of the double helix (one drug molecule per strand), thereby stabilizing cleavage complexes, inhibiting enzyme function, and increasing levels of gyrase/topoisomerase IV-mediated double-stranded DNA breaks [2,4,5,6,7,27]. When stabilized cleavage complexes are approached by replication forks, transcription complexes, and other systems that require opening of the double helix, they disrupt the ability of the enzymes to ligate the DNA [6,25,28]. This requires the breaks to be resected via recombination/repair pathways [6,7,25,28]. When these breaks reach a critical level, they activate SOS pathways and ultimately lead to cell death [29,30,31].

The most important mechanism of fluoroquinolone resistance is target-mediated and is caused by mutations in a highly conserved serine residue (originally described as Ser83 in the GyrA subunit of *Escherichia coli* gyrase) and an acidic residue four amino acids away at position 87 [2,4,5,6,7,32]. These residues interact with two water molecules that are coordinated by a metal ion that is chelated to the fluoroquinolone C3-C4 keto acid group [4,6,27,33,34]. This “water-metal ion bridge” is the main conduit for interactions between fluoroquinolones and gyrase/topoisomerase IV [4,6,27,33,34].

To address fluoroquinolone resistance, new classes of compounds are under development that also target gyrase/topoisomerase IV but interact with different amino acid residues in the enzymes [2,6,35,36,37,38]. The most clinically advanced class of new compounds are Novel Bacterial Topoisomerase Inhibitors (NBTIs) [2,6,39,40,41,42,43]. Phase 3 clinical trials for the treatment of uncomplicated urinary tract infections (caused by uropathogens, including *E. coli*) with a member of this class were stopped early because of demonstrated efficacy [39,41,43,44]. An NBTI is also in phase 3 trials for uncomplicated urogenital gonorrhea (*N. gonorrhoeae*) [40,42]. In contrast to fluoroquinolones, only a single NBTI molecule binds per cleavage complex [35,37,45]. The left-hand side of the molecule sits in a pocket in the DNA on the two-fold axis of the complex, midway between the two DNA cleavage sites, and the right-hand side sits in a pocket on the two-fold axis between the two GyrA/ParC subunits [35,37,45]. While fluoroquinolones induce gyrase/topoisomerase IV-mediated double-stranded breaks [4,6,7,27,46,47], a hallmark of NBTIs is the generation of enzyme-mediated single-stranded DNA breaks and the suppression of double-stranded breaks [37,48,49]. However, some dioxane-linked amide members of the NBTI class have been shown to induce double-stranded and single-stranded DNA breaks mediated by *Staphylococcus aureus* gyrase [50].

To further explore the ability of NBTIs to induce double-stranded DNA breaks mediated by bacterial type II topoisomerases, we examined the effects of OSUAB-185 (Figure 1) on the DNA cleavage activity of *N. gonorrhoeae* gyrase and topoisomerase IV. Although a member of the NBTI class, the substituents on this dioxane-linked amide NBTI differ considerably from those of canonical members. Similar to other NBTIs, OSUAB-185 induced single-stranded and suppressed double-stranded DNA breaks mediated by *N. gonorrhoeae* gyrase. However, in contrast to NBTIs such as GSK126 [49], OSUAB-185 stabilized both single- and double-stranded DNA breaks mediated by topoisomerase IV. It does not appear that the induction of double-stranded breaks is due to the binding of a second NBTI molecule. The ability to induce double-stranded DNA breaks extends to fluoroquinolone-resistant *N. gonorrhoeae* topoisomerase IV, as well as type II enzymes from other species. Together with previous work on the dioxane-linked amide NBTIs [50], the double-stranded DNA cleavage activity of OSUAB-185 represents a paradigm shift in the hallmark characteristic of NBTIs and suggests that some members of this class may have alternative binding motifs in the cleavage complex.

## 2. Results

### 2.1. OSUAB-185 Induces Double-Stranded DNA Cleavage Mediated by N. gonorrhoeae Topoisomerase IV

**Figure 2:**
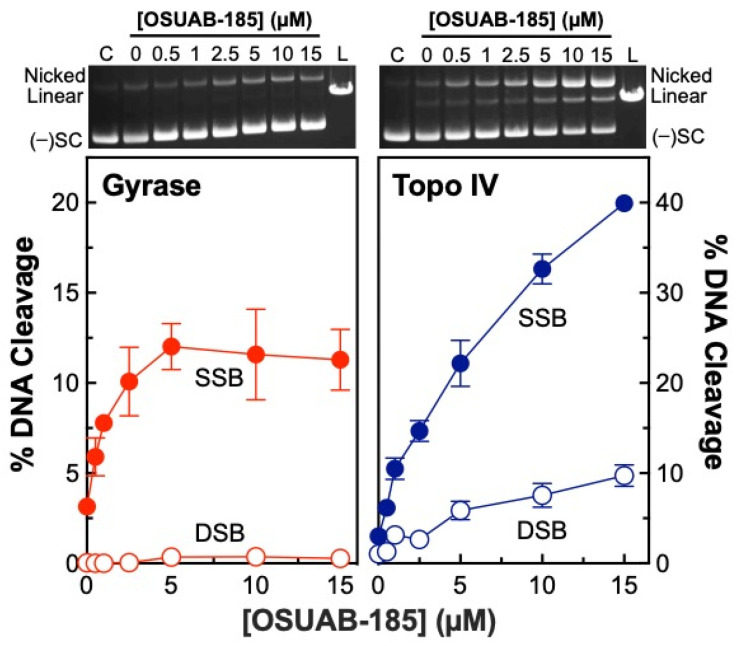
OSUAB-185 enhances DNA cleavage mediated by *N. gonorrhoeae* gyrase and topoisomerase IV. The graphs show the effects of OSUAB-185 on single-stranded (SSB, closed circle) and double-stranded (DSB, opened circle) DNA cleavage mediated by *N. gonorrhoeae* gyrase (red, (**left**)) and topoisomerase IV (Topo IV, blue, (**right**)). Note that the scaling of percent DNA cleavage on the y-axis differs between gyrase and topoisomerase IV. Error bars represent SDs for at least three independent experiments. Representative agarose gels for DNA cleavage assays with gyrase (**left**) and topoisomerase IV (**right**) are shown above the graphs. Control DNA (C) in the absence of enzyme and a linear DNA standard (L) are indicated. The positions of negatively supercoiled [(−)SC], nicked, and linear plasmid are shown.

A previous study demonstrated that some dioxane-linked NBTIs were able to induce double-stranded (in addition to single-stranded) DNA breaks mediated by *S. aureus* gyrase [50]. Therefore, to further explore the ability of this NBTI subclass to induce double-stranded DNA breaks generated by bacterial type II topoisomerases and to address the basis for this activity, we assessed the effects of OSUAB-185 on DNA cleavage mediated by *N. gonorrhoeae* gyrase and topoisomerase IV (Figure 2). The NBTI displayed moderate activity against gyrase, increasing single-stranded DNA breaks to ~12.0% at 5 µM compound (compared to a baseline level of ~3.1%) with a CC_50_ value (concentration of compound required to induce 50% of maximal DNA cleavage) of ~0.9 µM. As reported previously for other NBTIs [37,48,49], induction of double-stranded DNA cleavage was not observed. Although OSUAB-185 was less potent against topoisomerase IV (CC_50_ ≈ 4.8 µM), it was considerably more efficacious, inducing ~40% single-stranded DNA breaks at 15 µM compound (compared to a baseline level of ~3.0%). Strikingly, OSUAB-185 also increased double-stranded DNA breaks mediated by topoisomerase IV. Approximately 10% double-stranded DNA breaks were observed at 15 µM compound (compared to a baseline level of ~1%) with a CC_50_ value of ~5.7 µM. The increase in double-stranded breaks is contrary to the hallmark of NBTIs, which is the induction of single-stranded DNA breaks by gyrase/topoisomerase IV and the suppression of enzyme-mediated double-stranded DNA breaks [37,48,49].

**Figure 3:**
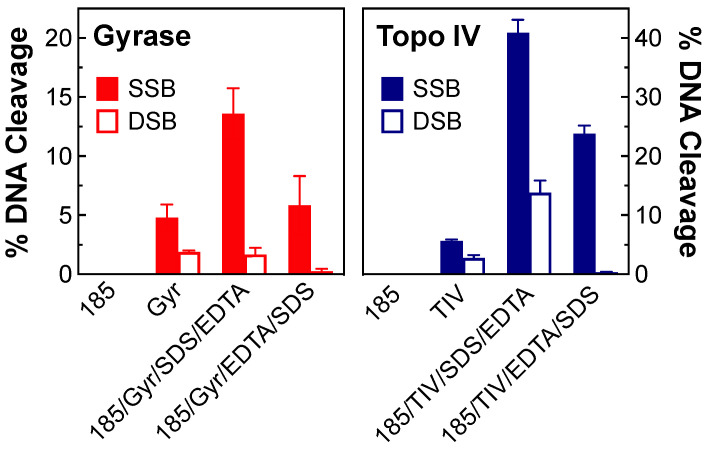
DNA cleavage induced by OSUAB-185 is medicated by *N. gonorrhoeae* gyrase and topoisomerase IV. Bar graphs show single-stranded (SSB, filled bars) and double-stranded (DSB, open bars) DNA cleavage mediated by *N. gonorrhoeae* gyrase (red, (**left**)) and topoisomerase IV (Topo IV, blue, (**right**)). Reactions contained negatively supercoiled DNA, with 10 μM OSUAB-185 in the absence of enzyme (185), gyrase (Gyr) or topoisomerase IV (TIV) in the absence of OSUAB-185, or complete reaction mixtures that contained enzyme, DNA, and the NBTI (185/Gyr/SDS/EDTA and 185/TIV/SDS/EDTA). All of these reactions were stopped with SDS prior to the addition of EDTA. Alternatively, reactions that contained enzyme, DNA, and the NBTI were treated with 2 µL of 250 mM EDTA for 10 min prior to the addition of SDS (185/Gyr/EDTA/SDS and 185/TIV/EDTA/SDS). Note that the scaling of percent DNA cleavage on the y-axis differs between gyrase and topoisomerase IV and that gyrase reactions contained 200 nM enzyme, which is twice the concentration used in other cleavage assays, to increase baseline levels of DNA cleavage. Error bars represent SDs for at least three independent experiments.

To ensure that the DNA breaks induced by OSUAB-185 were being generated by gyrase and topoisomerase IV, two controls were carried out (Figure 3). First, no single- or double-stranded DNA cleavage was observed in the presence of 10 µM compound in the absence of enzyme. Second, when DNA cleavage reactions were terminated by the addition of EDTA prior to SDS, DNA cleavage levels dropped for both enzymes. EDTA chelates the required catalytic Mg^2+^ ion, but only when the DNA is ligated [51]. Therefore, the decrease in cleaved DNA following EDTA treatment is inconsistent with a non-enzymatic reaction. These data provide strong evidence that the DNA breaks observed in the presence of OSUAB-185 were generated by gyrase and topoisomerase IV. It is notable that all of the NBTI-induced double-stranded breaks generated by topoisomerase IV disappeared in the presence of EDTA. This finding suggests that they may be less stable than the single-stranded DNA breaks generated in the presence of the compound.

**Figure 4:**
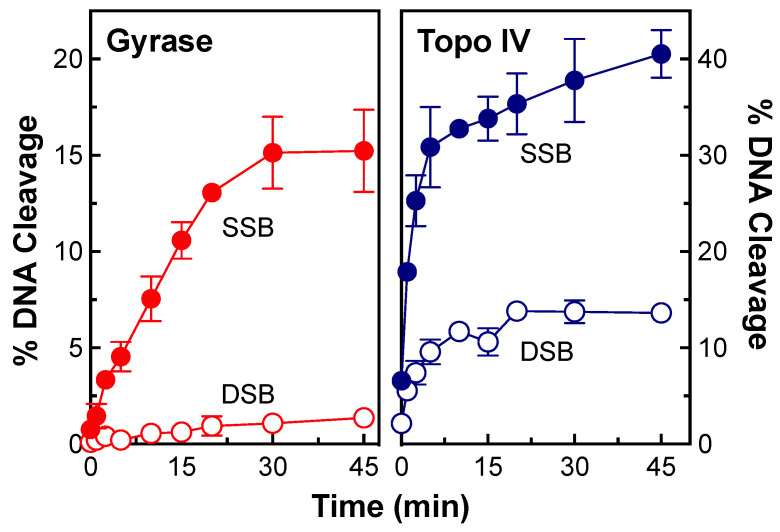
Time courses for DNA cleavage mediated by *N. gonorrhoeae* gyrase and topoisomerase IV. Time courses for single-stranded (SSB, closed circle) and double-stranded (DSB, opened circle) DNA cleavage mediated by gyrase (red, (**left**)) and topoisomerase IV (Topo IV, blue, (**right**)) in the presence of 10 μM OSUAB-185 are shown. Error bars represent SDs for at least three independent experiments.

**Figure 5:**
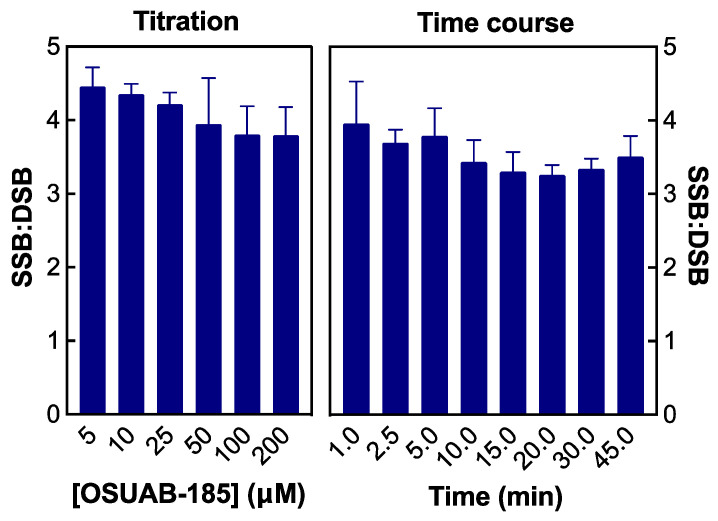
Ratios of NBTI-induced single-stranded to double-stranded DNA cleavage mediated by *N. gonorrhoeae* topoisomerase IV are maintained over OSUAB-185 concentrations and reaction time. The ratios of single-stranded to double-stranded (SSB:DSB) DNA breaks over a concentration range of OSUAB-185 ((**left**) panel) or a time course of DNA cleavage ((**right**) panel) induced by 10 µM NBTI with topoisomerase IV are shown. Error bars represent SDs for at least three independent experiments.

To determine whether the relative levels of topoisomerase IV-mediated double- vs. single-stranded DNA breaks change with time, a time course for DNA scission with both enzymes was monitored (Figure 4). As with the NBTI titration, no appreciable double-stranded breaks were observed with gyrase (Figure 4, left panel). In contrast, double- and single-stranded DNA breaks were observed with topoisomerase IV and appeared to be generated coordinately (Figure 4, right panel). To analyze the formation of double-stranded DNA breaks by topoisomerase IV in greater detail, data from the [OSUAB-185] titration (Figure 2, as well as NBTI concentrations up to 200 µM) and the time course for cleavage (Figure 4) were converted to ratios of single-stranded:double-stranded DNA breaks (Figure 5, left and right panel, respectively). The single-stranded:double-stranded DNA break ratios remained constant over a wide range of NBTI concentrations and cleavage reaction times. These findings provide further evidence that the single- and double-stranded DNA breaks generated by *N. gonorrhoeae* topoisomerase IV in the presence of OSUAB-185 were generated coordinately. This result argues against a second NBTI molecule entering the active site of topoisomerase IV at higher concentrations of OSUAB-185 or over longer reaction times. It also argues against a change in the binding conformation of the NBTI over time.

**Figure 6:**
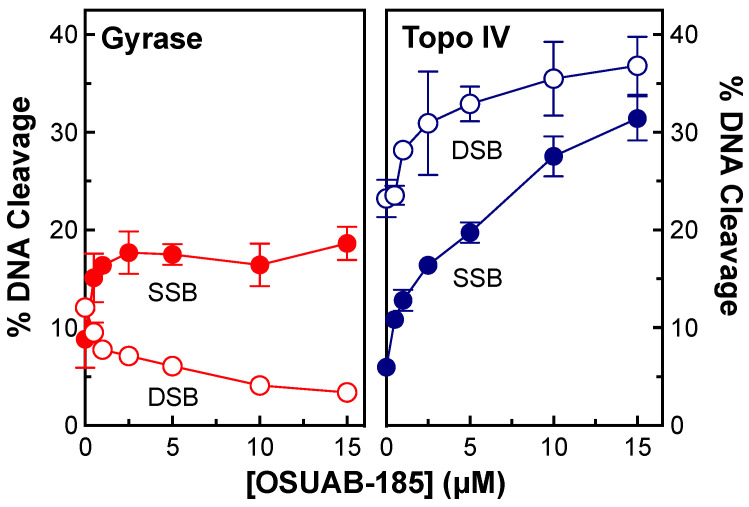
OSUAB-185 suppresses levels of double-stranded DNA cleavage mediated by *N. gonorrhoeae* gyrase and increases levels of double-stranded DNA breaks generated by topoisomerase IV. DNA cleavage reactions contained CaCl_2_ in place of MgCl_2_ to raise baseline levels of DNA scission. Graphs show the effects of OSUAB-185 on single-stranded (SSB, closed circle) and double-stranded (DSB, opened circle) DNA cleavage mediated by gyrase (red, (**left**)) and topoisomerase IV (Topo IV, blue, (**right**)). Error bars represent SDs for at least three independent experiments.

As a final demonstration that the induction of topoisomerase IV-mediated double-stranded DNA breaks by OSUAB-185 represents a novel mechanism of action, a titration of the NBTI was carried out in cleavage assays that replaced MgCl_2_ with CaCl_2_ (Figure 6). With some enzymes, Ca^2+^ dramatically raises baseline levels of single- and double-stranded DNA breaks, which affords a more ready assessment of NBTI effects on DNA cleavage [34,48,49,52]. Similar to previous results with other NBTIs, OSUAB-185 induced single-stranded DNA breaks and suppressed double-stranded breaks mediated by *N. gonorrhoeae* gyrase (Figure 6, left panel). However, in marked contrast, the NBTI enhanced both single- and double-stranded DNA cleavage mediated by topoisomerase IV (Figure 6, right panel). This finding provides strong evidence that OSUAB-185 induces double-stranded DNA breaks mediated by *N. gonorrhoeae* topoisomerase IV.

### 2.2. Stability of Single- and Double-Stranded DNA Breaks Induced by OSUAB-185

**Figure 7:**
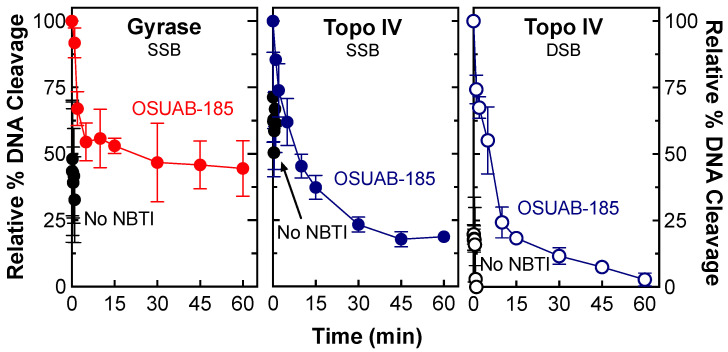
OSUAB-185 induces stable DNA cleavage complexes with *N. gonorrhoeae* gyrase and topoisomerase IV. Persistence reactions were allowed to reach cleavage–ligation equilibrium before dilution in reaction buffer that lacked MgCl_2_. The subsequent stability of cleavage complexes was monitored. Persistence of single-stranded DNA (SSB, closed circle) cleavage complexes by gyrase (red, (**left**) panel) and topoisomerase IV (blue, (**middle**) panel) and double-stranded DNA (DSB, open circle) cleavage complexes by topoisomerase IV (blue, (**right**) panel) formed in the presence of 10 μM OSUAB-185 are shown. Persistence reactions carried out in the absence of the NBTI (No NBTI) are shown in black (SSB, closed circle; DSB, open circle). DNA cleavage prior to dilution of cleavage complexes was set to 100%. Error bars represent SDs of three independent experiments.

Other things being equal, drugs that generate the most stable cleavage complexes appear to be the most lethal in cells [53]. Therefore, two approaches were utilized to assess the effects of OSUAB-185 on the stability of cleavage complexes formed by *N. gonorrhoeae* gyrase and topoisomerase IV. In the first approach, the effects of OSUAB-185 on the persistence of cleavage complexes were determined. In this assay, cleavage complexes were formed in the presence of high concentrations of enzyme and DNA, and the lifetimes of cleavage complexes were monitored following 20-fold dilution into reaction buffer that lacked the catalytic divalent metal ion. While the shift in condition does not alter the DNA cleavage–ligation equilibrium in established cleavage complexes, complexes that disassociate are unlikely to reform. As seen in Figure 7, cleavage complexes formed with gyrase (monitoring single-stranded DNA cleavage, left panel) or topoisomerase IV (monitoring single-stranded DNA cleavage, middle panel, or double-stranded DNA cleavage, right panel) in the absence of the NBTI were highly unstable and rapidly disassociated following dilution. Complexes became considerably more stable in the presence of OSUAB-185. Consistent with the data from Figure 3, NBTI-induced double-stranded DNA breaks (t_1/2_ ≈ 4 min) were less stable than single-stranded DNA breaks (t_1/2_ ≈ 8 min) with topoisomerase IV.

**Figure 8:**
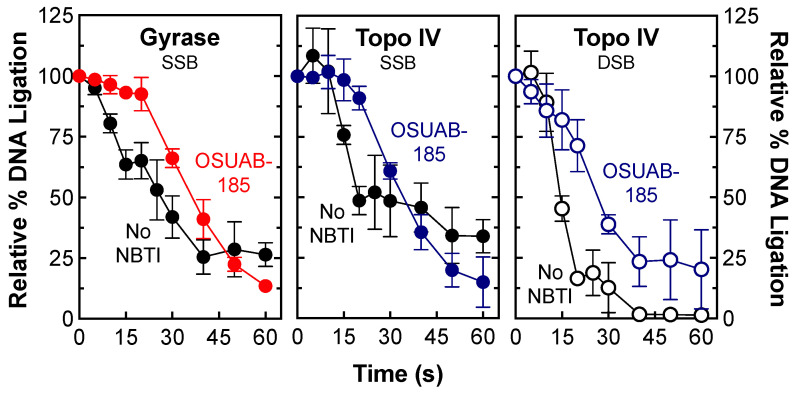
OSUAB-185 inhibits DNA ligation mediated by *N. gonorrhoeae* gyrase and topoisomerase IV. Ligation of single-stranded DNA (SSB, closed circle) cleavage complexes by gyrase (red, (**left**) panel) and topoisomerase IV (blue, (**middle**) panel) and double-stranded DNA (DSB, open circle) cleavage complexes by topoisomerase IV (blue, (**right**) panel) formed in the presence of 10 μM OSUAB-185 are shown. DNA ligation was also monitored in the absence of the NBTI (No NBTI, black, SSB, closed circle; DSB, open circle). Levels of single- and double-stranded DNA cleavage prior to the induction of ligation were set to 100%. Error bars represent SDs for at least three independent experiments.

In the second approach, the effects of OSUAB-185 on the rate of gyrase/topoisomerase IV-mediated DNA ligation were monitored by shifting cleavage complexes from 37 °C to 65 °C (a temperature that allows ligation but not cleavage of the DNA) [54]. As seen in Figure 8, OSUAB-185 had a modest effect on rates of ligation for single-stranded DNA breaks with gyrase (left panel) and topoisomerase IV (middle panel) and double-stranded breaks with topoisomerase IV (right panel). The NBTI generated slightly more stable single-stranded (t_1/2_ ≈ 35 s) DNA cleavage complexes than double-stranded (t_1/2_ ≈ 27 s) DNA cleavage complexes with topoisomerase IV.

### 2.3. Effects of OSUAB-185 on Fluoroquinolone-Resistant N. gonorrhoeae Gyrase and Topoisomerase IV

**Figure 9:**
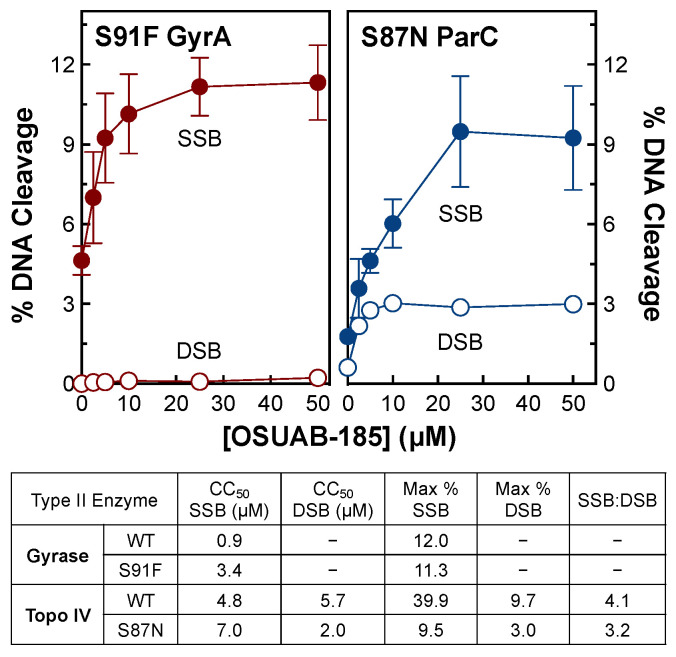
Activity of OSUAB-185 against *N. gonorrhoeae* gyrase and topoisomerase IV that contain fluoroquinolone-resistance mutations. The effects of OSUAB-185 on single-stranded (SSB, closed circle) and double-stranded (DSB, open circle) DNA cleavage mediated by fluoroquinolone-resistant GyrA^S91F^ gyrase (S91F GyrA; (**top left**), red) and ParC^S87N^ topoisomerase IV (S87N ParC; (**top right**), blue) are shown. Error bars represent SDs of three independent experiments. A summary table of DNA cleavage mediated by WT and mutant *N. gonorrhoeae* gyrase and topoisomerase IV is shown at the bottom. CC_50_ values are indicated for the enhancement of single-stranded and double-stranded DNA cleavage. Maximal levels of NBTI-induced DNA scission (Max %) are also shown. Significant levels of double-stranded DNA breaks were not observed with WT or GyrA^S91F^ gyrase. The ratio of single-stranded to double-stranded DNA breaks (SSB:DSB) were calculated at maximal levels of DNA cleavage for WT and ParC^S87N^ topoisomerase IV.

To determine whether OSUAB-185 is able to overcome fluoroquinolone resistance in *N. gonorrhoeae* type II enzymes, the effects of the NBTI on gyrase that contained GyrA^S91F^ and topoisomerase IV that contained ParC^S87N^ were examined. These two amino acid substitutions represent the most prevalent fluoroquinolone-resistant mutations in *N. gonorrhoeae* gyrase and topoisomerase IV, respectively (Figure 9) [55,56]. Levels of DNA cleavage with GyrA^S91F^ gyrase (top left panel) were similar to those obtained with the wild-type enzyme and only a single-stranded DNA cleavage was induced by OSUAB-185. However, the NBTI was ~4-fold less potent against the GyrA^S91F^ mutant. In contrast, levels of NBTI-induced DNA scission with ParC^S87N^ topoisomerase IV (top right panel) were ~3 to 4-fold lower than observed for the wild-type enzyme, and OSUAB-185 maintained its potency. Once again, the NBTI induced single- and double-stranded DNA scission with fluoroquinolone-resistant topoisomerase IV. These findings predict that the NBTI would retain at least some activity against fluoroquinolone-resistant *N. gonorrhoeae* cells harboring common mutations in gyrase/topoisomerase IV. They also suggest that the fluoroquinolone-resistant mutations do not alter the mechanism of action of OSUAB-185 against bacterial type II topoisomerases.

### 2.4. Effects of OSUAB-185 on Gyrase and Topoisomerase IV from E. coli and S. aureus

**Figure 10:**
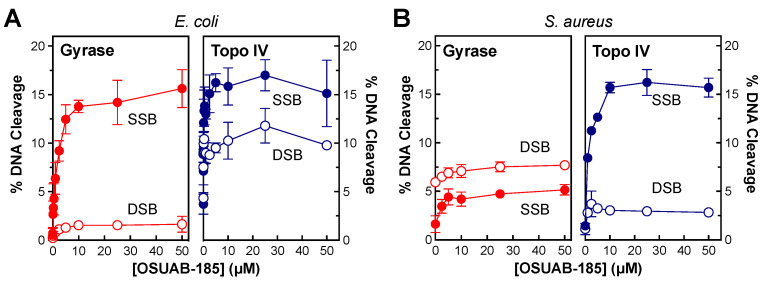
OSUAB-185 induces double-stranded DNA breaks mediated by gyrase and topoisomerase IV from *E. coli* and *S. aureus*. The effects of OSUAB-185 on single-stranded (SSB, closed circles) and double-stranded (DSB, open circles) DNA cleavage mediated by gyrase (red, left panels) and topoisomerase IV (blue, right panels) from *E. coli* (**A**) and *S. aureus* (**B**) are shown. Error bars represent SDs of three independent experiments.

To determine whether OSUAB-185 is able to induce double-stranded DNA breaks with type II topoisomerases from other species (Figure 10), the effects of the NBTI on DNA cleavage mediated by gyrase (left panels) and topoisomerase IV (right panels) from *E. coli* (Figure 10A) and *S. aureus* (Figure 10B) were assessed. Note that the induction of double-stranded DNA breaks by OSUAB-185 and *S. aureus* gyrase has been reported previously [50]. To at least some extent, OSUAB-185 induced double-stranded breaks with all four of the enzymes. Double-stranded DNA breaks were especially prominent with *E. coli* topoisomerase IV. These data indicate that the ability of the NBTI to generate enzyme-mediated double-stranded DNA breaks is not confined to *N. gonorrhoeae* or either type II topoisomerase.

### 2.5. Effects of OSUAB-185 on Human Topoisomerase IIα

**Figure 11:**
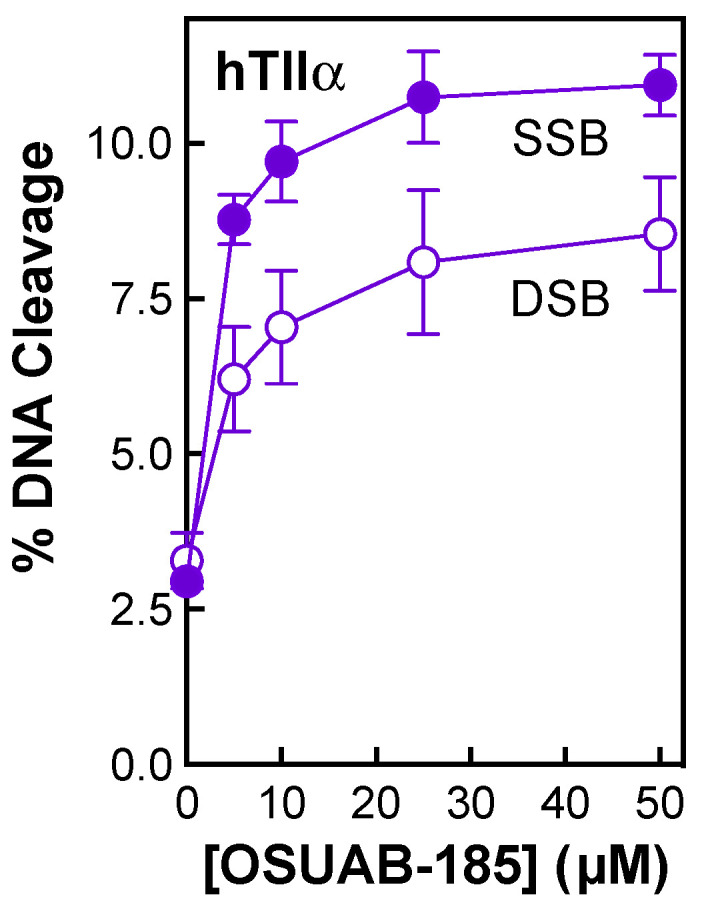
Effects of OSUAB-185 on DNA cleavage mediated by human topoisomerase IIα. The graph shows the effects of OSUAB-185 on single-stranded (SSB, purple, closed circle) and double-stranded (DSB, purple, open circle) DNA cleavage mediated by human topoisomerase IIα. Error bars represent standard error of the mean of two independent experiments.

Very little is known about the interaction of NBTIs with human type II topoisomerases [57,58,59]. Therefore, the effects of OSUAB-185 on DNA cleavage mediated by human topoisomerase IIα was determined (Figure 11). The NBTI displayed reasonable activity against the human type II enzyme, with CC_50_ values in the low µM range. In addition, similar to the results with some of the bacterial enzymes, OSUAB-185 induced moderate levels of double-stranded DNA breaks with the human enzyme (maximal levels of cleavage of ~11% and ~8.5% for single- and double-stranded DNA breaks, respectively). This last finding provides further evidence that some members of the NBTI class are capable of generating enzyme-mediated double-stranded DNA breaks.

## 3. Discussion

NBTIs are an emerging class of compounds with antibacterial activity. In contrast to fluoroquinolones, a hallmark of most NBTIs is their ability to induce gyrase/topoisomerase IV-mediated single-stranded breaks in DNA and suppress the formation of double-stranded breaks [37,48,49]. Together with a previous study, the present work provides strong evidence that select dioxane-linked amide NBTIs are also capable of generating double-stranded DNA breaks mediated by type II topoisomerases from a variety of bacterial species, as well as humans [50].

Fluoroquinolones induce double-stranded DNA breaks because two drug molecules bind in the active site of gyrase and topoisomerase IV, with one molecule stabilizing a cleaved scissile bond on each strand of the double helix [27,46,47]. In contrast, only a single NBTI molecule binds in the active site of the bacterial type II topoisomerases, with the molecule binding midway between the two scissile bonds [35,37,45]. It is believed that canonical NBTIs stabilize single-stranded and suppress double-stranded DNA breaks by distorting the active site of gyrase/topoisomerase IV in a manner that inhibits ligation of the first strand but does not allow cleavage of the second [35,37]. It is not known how OSUAB-185 and potentially other dioxane-linked amide NBTIs generate double-stranded breaks. Results from OSUAB-185 titrations (Figure 2 and Figure 5) and time course experiments (Figure 4 and Figure 5) strongly suggest that it is not due to the binding of a second molecule of compound. Potentially, this NBTI has two mutually exclusive binding configurations that distort the active site of the enzyme differently. In the first, the NBTI acts as a canonical member of this class and induces distortion after one strand is cut to inhibit ligation and prevent cleavage of the second strand (thus enhancing single-stranded DNA breaks). In the second, the NBTI acts in a non-canonical manner and only induces the dramatic active site distortion after both DNA strands are cleaved (thus enhancing double-stranded DNA breaks). Future structural studies are needed to determine if this is the case.

The activity of OSUAB-185 against topoisomerase IIα suggests that this member of this NBTI subclass might cross over to human systems if used to treat infections. However, our results raise an interesting possibility. The double-stranded DNA breaks generated by human type II topoisomerases during transcription have the potential to trigger secondary leukemias in a small percentage of patients treated with topoisomerase II-targeted anticancer drugs [25,60,61,62,63]. If a drug was developed that induced only single-stranded DNA breaks with human type II topoisomerases, it is possible that it could overcome the secondary leukemias observed with current drugs. Although OSUAB-185 induced both single- and double-stranded DNA breaks mediated by human topoisomerase IIα, our findings open the door for future work on NBTIs and human type II topoisomerases.

## 4. Materials and Methods

### 4.1. Enzymes and Materials

Wild-type *N. gonorrhoeae* gyrase (GyrA, GyrB) and topoisomerase IV (ParC, ParE) subunits were prepared as described previously [35,46]. Fluoroquinolone-resistant *N. gonorrhoeae* GyrA^S91F^ was a gift from Dr. Pan Chan (GlaxoSmithKline, Brentford, UK) and fluoroquinolone-resistant *N. gonorrhoeae* ParC^S87N^ was generated using a QuickChange II XL site-directed mutagenesis kit (Agilent Technologies, Santa Clara, CA, USA) with custom primers for the desired mutations. Mutant *N. gonorrhoeae* ParC^S87N^ was expressed and purified as described by Ashley et al. [64] with the following modifications to optimize protein expression and lysis: (1) ParC^S87N^ was expressed for 3 h before harvesting, and (2) cells were lysed by sonication using a digital sonifier (Branson, Danbury, CT, USA). Wild-type *E. coli* gyrase (GyrA, GyrB) subunits were expressed and purified as described by Chan et al. [46], and wild-type *E. coli* topoisomerase IV (ParC, ParE, a gift from Dr. Keir Neumann, NHLBI), subunits were expressed and purified as described by Peng and Marians [65]. Wild-type *S. aureus* gyrase (GyrA, GyrB) and topoisomerase IV (GrlA and GrlB) subunits were ordered from GenScript and expressed and purified as described previously [37,46]. Recombinant human wild-type topoisomerase IIα was expressed in *Saccharomyces cerevisiae* and purified as described previously [66,67]. The identities of all constructs were confirmed by DNA sequencing, and all enzymes were stored at −80 °C.

Negatively supercoiled pBR322 DNA was prepared from *E. coli* using a Plasmid Mega Kit (Qiagen, Hilden, Germany) as described by the manufacturer. OSUAB-185 was synthesized as described previously [50] (shown as compound 23) and stored at 4 °C as a 20 mM stock solution in 100% DMSO. All other chemicals were of analytical reagent grade.

### 4.2. DNA Cleavage

DNA cleavage reactions were performed according to the procedure of Aldred et al. [33] (gyrase and topoisomerase IV) or Fortune and Osheroff [68] (human topoisomerase IIα). Reactions were performed in the absence of an NBTI or in the presence of increasing concentrations of OSUAB-185. Unless stated otherwise, assay mixtures contained 10 nM pBR322 and 100 nM wild-type or mutant (GyrA^S91F^) *N. gonorrhoeae* gyrase, 100 nM wild-type or mutant (ParC^S87N^) *N. gonorrhoeae* topoisomerase IV, 100 nM wild-type *E. coli* gyrase, 20 nM wild-type *E. coli* topoisomerase IV, 100 nM wild-type *S. aureus* gyrase, 20 nM *S. aureus* topoisomerase IV, or 300 nM wild-type human topoisomerase IIα in a total volume of 20 µL of cleavage buffer: 40 mM Tris-HCl (pH 7.9), 50 mM NaCl, 2.5% (*w*/*v*) glycerol, and 5 mM or 10 mM MgCl_2_ for *E. coli* or *N. gonorrhoeae* type II topoisomerases, respectively; 50 mM Tris-HCl (pH 7.5), 100 mM KGlu, 5 mM MgCl_2_, 5 mM dithiothreitol (DTT), and 50 μg/mL BSA for *S. aureus* gyrase; 40 mM Tris-HCl (pH 7.5), 6 mM MgCl_2_, 20 mM NaCl, 10 mM DTT, and 50 μg/mL BSA for *S. aureus* topoisomerase IV; 50 mM Tris-HCl (pH 7.9), 0.5 mM EDTA (pH 8.0), 500 mM KCl, 25 mM MgCl_2_, and 12.5% (*w*/*v*) glycerol for human topoisomerase IIα. In some cases, MgCl_2_ in the cleavage buffer was replaced with an equivalent concentration of CaCl_2_. Reactions were incubated at 37 °C for 30 min for wild-type and mutant (GyrA^S91F^) *N. gonorrhoeae* gyrase, mutant (ParC^S87N^) *N. gonorrhoeae* topoisomerase IV, and wild-type *S. aureus* gyrase; 20 min with wild-type *E. coli* gyrase; and 10 min for all other enzymes. Enzyme–DNA cleavage complexes were trapped by adding 2 µL of 4% SDS, followed by 2 µL of 250 mM EDTA (pH 8.0). Proteinase K was added (2 µL of a 0.8 mg/mL solution), and reaction mixtures were incubated for 30 min at 45 °C to digest the enzyme. Samples were mixed with 2 µL of agarose loading buffer [60% sucrose, 10 mM Tris-HCl (pH 7.9), 0.5% bromophenol blue, and 0.5% xylene cyanol FF] and heated for 2 min at 45 °C prior to electrophoresis in 1% agarose gels in 40 mM Tris-acetate (pH 8.3), and 2 mM EDTA containing 0.5 µg/mL ethidium bromide. DNA bands were visualized by mid-range ultraviolet light and quantified using an Alpha Innotech digital imaging system (Protein Simple, San Jose, CA, USA). DNA cleavage was monitored by the conversion of negatively supercoiled plasmid DNA to nicked (single-stranded break) or linear (double-stranded break) molecules. CC_50_ values (the concentration of compound that induced 50% maximal DNA cleavage complex formation) were calculated on GraphPad Prism Version 9.5.1 using a non-linear regression analysis with 95% confidence intervals.

### 4.3. Persistence of Cleavage Complexes

The persistence of gyrase/topoisomerase IV–DNA cleavage complexes was determined as described previously [33]. Cleavage complexes were formed by combining 50 nM pBR322 and 200 nM *N. gonorrhoeae* gyrase or 100 nM *N. gonorrhoeae* topoisomerase IV in the presence of 10 µM OSUAB-185 in a total volume of 20 µL of a cleavage buffer. Parallel control experiments were conducted to assess cleavage complexes formed in the absence of the NBTI by combining 50 nM pBR322 and 500 nM *N. gonorrhoeae* gyrase/topoisomerase IV in a 20 µL cleavage buffer. Reactions were incubated at 37 °C until the DNA cleavage/ligation equilibria were reached (30 min with gyrase and 10 min with topoisomerase IV) and diluted 20-fold in a DNA cleavage buffer lacking Mg^2+^. Samples (20 µL) were removed at time points ranging from 0 to 60 min, and reactions were stopped with 2 µL of 4% SDS, followed by 2 µL of 250 mM EDTA (pH 8.0). Proteinase K was added (2 µL of a 0.8 mg/mL solution), and the reactions mixtures were incubated for 30 min at 45 °C. Samples were mixed with 2 µL of agarose gel loading buffer and processed and analyzed as described above. Levels of DNA cleavage were set to 100% at time zero, and the persistence of cleavage complexes were determined by the decay of nicked (single-stranded breaks) or linear (double-stranded breaks) reaction products over time. Cleavage complex stability (half-life, t_1/2_) was calculated on GraphPad Prism Version 9.5.1 using non-linear regression analysis with 95% confidence intervals.

### 4.4. DNA Ligation

DNA ligation mediated by *N. gonorrhoeae* gyrase and topoisomerase IV was monitored using the procedure of Aldred et al. [33]. Initial reactions contained 10 nM pBR322 and 100 nM wild-type *N. gonorrhoeae* gyrase or topoisomerase IV in the absence or presence of 10 µM OSUAB-185. In order to better visualize levels of DNA breaks mediated by gyrase or topoisomerase IV in the absence of the NBTI, 10 mM MgCl_2_ was replaced by 10 mM CaCl_2_. DNA cleavage/ligation equilibria were established for 30 min with gyrase and 10 min with topoisomerase IV at 37 °C. DNA ligation was initiated by shifting samples from 37 °C to 65 °C, which allows enzyme-mediated ligation but prevents new rounds of DNA cleavage from occurring [54]. This results in a unidirectional sealing of the cleaved DNA. Reactions were stopped at time points ranging from 0 to 60 s by the addition of 4% SDS, followed by 2 µL 250 mM EDTA (pH 8.0). Proteinase K was added (2 µL of a 0.8 mg/mL solution), and reaction mixtures were incubated for 30 min at 45 °C. Samples were mixed with 2 µL of agarose loading buffer and processed and analyzed as described above. Nicked and linear DNA cleavage products at time zero were set to 100% to allow direct comparison between different conditions, and DNA ligation of single- or double-stranded breaks was monitored by the loss of nicked or linear DNA, respectively. The rate of DNA ligation (t_1/2_) was calculated on GraphPad Prism Version 9.5.1 using non-linear regression analysis with 95% confidence intervals.

### 4.5. Accession Codes

*N. gonorrhoeae* Gyrase, GyrA: UniProtKB P48371, GyrB: UniProtKB P22118; *N. gonorrhoeae* Topoisomerase IV, ParC: UniProtKB P48374, ParE: UniProtKB A0A8D9YA86; *E. coli* Gyrase, GyrA: UniProtKB P0AES4, GyrB: UniProtKB P0AES6; *E. coli* Topoisomerase IV, ParC: UniProtKB P0AFI2, ParE: UniProtKB P20083; *B. anthracis* Gyrase, GyrA: UniProtKB A0A0F7R8R3, GyrB: UniProtKB Q9X3Y6; *B. anthracis* Topoisomerase IV, GrlA: UniProtKB A0A6H3AG07, GrlB: UniProtKB A0A2B0YAF3; *S. aureus* Gyrase, GyrA: UniProtKB Q2FKQ0, GyrB: UniProtKB P0A0K8; *S. aureus* Topoisomerase IV, GrlA: UniProtKB P0C1U9, GrlB: UniProtKB P0C1S7; Human Topoisomerase IIα: UniProtKB P11388.

## Data Availability

All data are contained within the article.
